# Current Efavirenz (EFV) or Ritonavir-Boosted Lopinavir (LPV/r) Use Correlates with Elevate Markers of Atherosclerosis in HIV-Infected Subjects in Addis Ababa, Ethiopia

**DOI:** 10.1371/journal.pone.0117125

**Published:** 2015-04-27

**Authors:** Rudolph L. Gleason, Alexander W. Caulk, Daniel Seifu, Ivana Parker, Brani Vidakovic, Helena Getenet, Getachew Assefa, Wondwossen Amogne

**Affiliations:** 1 The George W. Woodruff School of Mechanical Engineering, Georgia Institute of Technology, Atlanta, GA, United States of America; 2 The Wallace H. Coulter Department of Biomedical Engineering, Georgia Institute of Technology, Atlanta, GA, United States of America; 3 The Petit Institute for Bioengineering and Bioscience, Georgia Institute of Technology, Atlanta, GA, United States of America; 4 Department of Biochemistry, Medical Faculty, Addis Ababa University, Addis Ababa, Ethiopia; 5 Department of Internal Medicine, Medical Faculty, Addis Ababa University, Addis Ababa, Ethiopia; 6 Department of Radiology, Medical Faculty, Addis Ababa University, Addis Ababa, Ethiopia; University of Toronto, CANADA

## Abstract

**Background:**

HIV patients on antiretroviral therapy have shown elevated incidence of dyslipidemia, lipodystrophy, and cardiovascular disease (CVD). Most studies, however, focus on cohorts from developed countries, with less data available for these co-morbidities in Ethiopia and sub-Saharan Africa.

**Methods:**

Adult HIV-negative (*n* = 36), treatment naïve (*n* = 51), efavirenz (EFV)-treated (*n* = 91), nevirapine (NVP)-treated (*n* = 95), or ritonavir-boosted lopinavir (LPV/r)-treated (*n*=44) subjects were recruited from Black Lion Hospital in Addis Ababa, Ethiopia. Aortic pressure, augmentation pressure, and pulse wave velocity (PWV) were measured via applanation tonometry and carotid intima-media thickness (cIMT) and carotid arterial stiffness, and brachial artery flow-mediated dilation (FMD) were measured via non-invasive ultrasound. Body mass index, waist-to-hip circumference ratio (WHR), skinfold thickness, and self-reported fat redistribution were used to quantify lipodystrophy. CD4+ cell count, plasma HIV RNA levels, fasting glucose, total-, HDL-, and LDL-cholesterol, triglycerides, hsCRP, sVCAM-1, sICAM-1, leptin and complete blood count were measured.

**Results:**

PWV and normalized cIMT were elevate and FMD impaired in EFV- and LPV/r-treated subjects compared to NVP-treated subjects; normalized cIMT was also elevated and FMD impaired in the EFV- and LPV/r-treated subjects compared to treatment-naïve subjects. cIMT was not statistically different across groups. Treated subjects exhibited elevated markers of dyslipidemia, inflammation, and lipodystrophy. PWV was associated with age, current EFV and LPV/r used, heart rate, blood pressure, triglycerides, LDL, and hsCRP, FMD with age, HIV duration, WHR, and glucose, and cIMT with age, current EFV use, skinfold thickness, and blood pressure.

**Conclusions:**

Current EFV- or LPV/r-treatment, but not NVP-treatment, correlated with elevated markers of atherosclerosis, which may involve mechanisms distinct from traditional risk factors.

## Background

People living with HIV taking highly active antiretroviral therapy (HAART) have shown elevated incidence of dyslipidemia, lipodystrophy, insulin resistance, diabetes mellitus, and cardiovascular disease (CVD)[1]; the latter includes higher prevalence of myocardial infarction [2,3] and atherosclerotic lesions [4], as well as elevated preclinical markers atherosclerosis including increased arterial stiffness [5,6] and carotid artery intima-media thickness (cIMT) [6,7], and impaired flow-mediated dilation (FMD) [8]. Of the five classes of antiretroviral drugs, protease inhibitors (PI) are the most widely implicated drug class in early on-set CVD, but nucleoside reverse transcriptase inhibitors (NRTI’s) and non-NRTI’s (NNRTI’s) have also been implicated [3,9–11].

A pressing need remains to better quantify non-AIDS related co-morbidities in people living with HIV in sub-Saharan Africa. Most studies of cardiovascular co-morbidities with HIV represent populations from developed countries; yet, 69% of all people living with HIV reside in sub-Saharan Africa [12]. Antiretroviral programs in Africa have made a tremendous impact in reducing HIV-related deaths and have been available in Ethiopia since 2005; the treatment disparities, however, between the developing world and the developed world remain great. People living with HIV in the developing world have access to fewer combinations of HAART regimens and patient adherence and clinical monitoring of CD4+ cell counts and viral loads in the developing world is well below that in the developed countries. As a result, people living with HIV in developing nations remain on first line therapy with less follow up. These treatment disparities, combined with differences in demographics, lifestyle, and nutritional status between Ethiopian and Western populations, may make this population more susceptible to non-AIDS related co-morbidities.

This paper reports a cross-sectional study to assess preclinical markers of atherosclerosis, lipodystrophy, dyslipidemia, and systemic inflammation with HIV infection and HAART in Addis Ababa, Ethiopia. We hypothesize that current efavirenz (EFV)- or ritonavir-boosted lopinavir (LPV/r)-treatment will correlate with increased arterial stiffness and cIMT and impaired FMD compared to current nevirapine (NVP)-treatment and no HAART-treatment, and that these preclinical markers will be associated with markers of lipodystrophy and inflammation.

## Materials and Methods

### Participant Enrollment and Baseline Data

All work was performed in accordance with the Declaration of Helsinki. All participants provided written informed consent and this study was approved by the Institutional Review Board Committees at Addis Ababa University and Georgia Institute of Technology. Eighteen- to 65-year-old HIV-negative, HIV-positive HAART naïve, and HIV-positive subjects on EFV-, NVP-, or LPV/r-containing regimens for at least two months prior to the exam were recruited from Tikur Anbessa (Black Lion) Specialized Referral Hospital in Addis Ababa, Ethiopia to participate in this study. Subjects were excluded if they had active AIDS defining illnesses or diabetes mellitus. Subjects fasted and refrained from tobacco products for at least 8 hours prior to the test and refrained from exercise in the morning of the test.

Participant age, sex, HIV-serostatus, date of first HIV-seropositive test, initial CD4+ cell count, last CD4+ cell count, and any viral load determinations, and date of initiation of current and all previous HAART regimens were obtained from the participants hospital card. Questionnaire-driven interviews were performed by the local recruiting nurse at the Black Lion Hospital HIV clinic under the direction of the research team. Self-reported personal and familial (mother, father, brothers, or sisters) history of heart attack, angina, stroke, kidney disease, diabetes, or lipid disorders and self-reported alcohol and cigarette use were recorded.

### Body Composition

Body weight, body height, waist and hip circumference, and skinfold thickness (bicep, tricep, suprailiac, and sub scapula) were measured. Skinfold measurements were taken in duplicate on the right and left sides and reported as the average of these four values. The ratio of the sum of skin-fold measurements in the suprailiac and sub-scapula divided by the sum of those from the bicep and tricep was reported as the Trunk/arm ratio. Subjects were asked if they observed an ‘increase in fat’, ‘decrease in fat’, or ‘no change’ at each of the following locations: face, arms, legs, abdomen, chest, cervical fat pad, supraclavicular fat, and across their entire body.

### Blood Pressure, Pulse Wave Analysis, and Pulse Wave Velocity

Brachial artery systolic (B-SBP) and diastolic (B-DBP) blood pressure were measured with a digital automatic blood pressure measurement device after the subjects rested in a supine position for at least 10 minutes. Pulse wave analysis (PWA) and pulse wave velocity (PWV) measurements were subsequently taken with a SphygmoCor CPV Clinical System (AtCor Medical) following manufacturer protocol. PWA and PWV measurements meeting the manufacturer quality control criteria were collected in triplicate and averaged.

### Carotid intima-media thickness (cIMT)

Ultrasound measurements were collected using a Sonocyte Micromax portable ultrasound device with vascular software for two-dimensional imaging, color and spectral Doppler, high- frequency (10 MHz) linear transducer, and internal electrocardiogram monitor. The right and left common carotid arteries (CCA) were examined with the head in the midline position tilted slightly upward and away from the artery being imaged. The probe was placed so that the near and far walls were parallel in the acquired image and lumen diameter was maximized in the longitudinal plane. Three 6-second clips of each artery were collected and stored for analysis. cIMT was reported in systole and diastole as the average of the near and far wall measurements from three separate clips from both the right and left CCA. In addition, given that clinically relevant differences in cIMT are on the order of microns and typical standard deviations observed across broad ranges of age and both sexes can be on the order of microns, mainly due to subject-to-subject variability in the carotid artery size, a normalized measure was defined as cIMT^norm^ = cIMT/(*D*/2), where *D* was the measured inner diameter of the CCA. It should be noted that this normalized value of cIMT is not been shown to be associated with cardiovascular risk; however, this measure allows for the normalization of data across a broad range of common carotid artery size.

The linearized cyclic strain Δε = Δ*D/D*, was also reported, where Δ*D* is the difference between the systolic and diastolic luminal diameter and *D* is the diastolic luminal diameter. The Peterson’s modulus (*E*_*p*_), a common measure of arterial stiffness, defined as *E*_*p*_ = *PP/*Δε = *1/C*, where *PP* is the pulse pressure and *C* is the compliance, was reported. The medial hoop stress approximated as σ_θ_ = *P*(*D*/2)/(cIMT) = *P*/cIMT^norm^, was also reported. *P* and *PP* in the carotid artery were approximated by the values determined for the aorta via PWA. A single analyst, blinded to the patients HIV status and HAART regimen, analyzed all images.

### Flow-mediated dilation

An appropriately sized sphygmomanometer was placed on the widest part of the proximal right forearm ~1 cm distal to the antecubital fossa. Prior to inflation, three 6-second clips of the brachial artery image and EKG tracings were stored for baseline measurements. The sphygmomanometer was inflated at least 50 mmHg above the subjects’ systolic blood pressure. Five minutes after occlusion, the cuff was deflated and a 6-second clip of the brachial artery image and EKG tracing was stored at 30, 45, 60, 75, and 90 seconds after deflation. A single analyst, blinded to the patients HIV status and HAART regimen, analyzed all images. Further, *FMD* = (*D*_*t*_—*D*_*B*_)/*D*_*B*_, where *D*_*t*_ is the brachial artery inner diameter during diastole at the time-point of interest and *D*_*B*_ is the average of the three measurements of the brachial artery diameter during diastole at baseline.

### Blood Sample Analysis

Complete blood count, total cholesterol (TC), triglyceride (TG), high-density lipoprotein cholesterol (HDL), low-density lipoprotein cholesterol (LDL), and glucose analysis were performed at the clinical laboratory at Black Lion Hospital. CD4+ cell count and plasma HIV RNA analysis was performed at the International Clinical Laboratory (Addis Ababa, Ethiopia). High-sensitivity C-reactive protein (hsCRP), soluble vascular cell adhesion molecule-1 (sVCAM-1) and intercellular adhesion molecule-1 (sICAM-1), and leptin analyses were performed on blood serum using commercially available ELISA kits (Life Technologies Corporation). Analysis for HIV viral load was performed on 70%, hsCRP on 80%, sVCAM-1 on 85%, sICAM-1 on 80%, and leptin on 80% of the subject pool, equally distributed among groups.

### Statistical Analysis

All statistical analyses were performed using MATLAB^©^ (MathWorks). A one-way analysis of variance (ANOVA) was performed to determine statistical significance across groups (*p* < 0.05) on continuous variables that satisfied Bartlett's test for equal variances and Pearson Chi-square test for normality of residuals; a Kruskal-Wallis non-parametric one-way ANOVA was performed on continuous variables that did not show equal variances or normality. For continuous variables with significance across groups, a pairwise Wilcoxon rank sum test was performed to detect differences between individual groups (*p* < 0.05). For categorical variables, χ^2^ analysis was performed to determine statistical significance across groups (*p* < 0.05); for those with significance across groups, a pairwise Marascuillo procedure was performed to compare differences in proportions between individual groups (*p* < 0.05).

Correlation analysis was performed and multivariable linear regression models were constructed to examine the association of study parameters with PWV for all HAART treated subjects. Relevant study parameters were selected for the inclusion of the initial models and the stepwisefit multivariable regression MATLAB subroutine, which eliminates (or adds) covariates in a stepwise fashion, was used with the criteria *p*<0.05 for removal from (or inclusion in) the final model.

## Results

### Participant Characteristics

CD4+ cell count was lower in all HIV-positive groups, compared to HIV-negative subjects, and lower in LPV/r-treated, compared to NVP-treated subjects (Table 1). Plasma HIV RNA levels were lower and percentages of subjects with plasma HIV RNA levels below detectable limits (<40 copies/mL) were higher in all HAART-treated groups, compared to HAART-naïve subjects. Years since first HIV diagnosis was higher in HAART-treated groups compared to HAART-naïve and in NVP- and LPV/r-treated compared to EFV-treated subjects. LPV/r-treated subjects were on HAART longer than EFV-treated subjects. EFV-treated subjects have been on their current regimen longer than LPV/r-treated, but shorter than NVP-subjects. The HAART treated groups had similar distributions of NRTI-backbone, with the exceptions that less LPV/r-treated subjects had an AZT-3TC backbone compared to NVP-treated subjects and more LPV/r-treated subjects had an NRTI-backbone other than AZT-3TC or TDF-3TC compared to EFV-treated subjects. The ‘Other’ NRTI backbones included stavudine in the NNRTI groups and didanosine, abacavir, or stavudine in the LPV/r group. More LPV/r-treated subjects had taken a previous regimen, which was expected, as LPV/r is considered a ‘second-line’ regimen. See S1 File for additional participant characteristics; namely, cigarette smoking, personal and family history of heart attack, angina, or stroke, kidney disease, diabetes, or lipid disorders, and complete blood count results.

**Table 1:** Baseline characteristics, body composition.

End-point	HIV-Negative	HAART Naïve	Efavirenz (EFV)	Nevarapine (NVP)	Lopinavir/r (LPV/r)
	**(n=36)**	**(n=51)**	**(n=91)**	**(n=95)**	**(n=44)**
**Demographics**	** **				
Age (yrs)	**39**(29-45)	**38**(32-45)	**38**(34-45)	**37**(32-42)	**39**(35-44)
Male ratio [# (%)]	**8** (22%)	**14**(27%)	**23** (25%)	**21**(22%)	**14** (32%)
**HIV / HAART History**					
CD4+ count	**730**(656-1047)	**395**(182-546)^A^	**349**(232-481)^A^	**390**(271-534)^A^	**285**(147-453)^A,e^
Viral Load (log10copies/mL)	**--**	**3.6**(2.2-4.7)	**<1.6**(<1.6-<1.6)^B^	**<1.6**(<1.6-<1.6)^B^	**<1.6**(<1.6-<1.6)^B^
Viral Load (% BDL)	**--**	**18%**	**92%B**	**87%** ^**B**^	**81%** ^**B**^
Yrs since diagnosis	**--**	**1.6**(0.3-3.7)	**5.7**(3.4-7.0)^B^	**6.0**(5.6-7.4)^B,d^	**6.5**(5.3-8.3)^B,d^
Yrs on HAART	**--**	--	**5.0**(3.2-6.2)	**5.7**(3.9-6.2)	**5.9**(4.2-6.8)^d^
Yrs on Current Regimen	**--**	--	**2.5**(1.5-4.4)	**3.8**(1.9-5.7)^d^	**1.8**(0.7-3.2)^E^
Current NRTI backbone					
TDF+3TC [# (%)]	**--**	--	**55**(60%)	**38**(40%)	**28**(64%)
AZT+3TC [# (%)]	**--**	--	**35**(38%)	**53**(56%)	**7**(16%)^E^
Other [# (%)]	**--**	--	**1**(1%)	**4**(4%)^e^	**9**(20%)
Previous Regimen					
All [# (%)]	**--**	--	**39**(43%)	**29**(31%)	**40**(91%)^D,E^
EFV-containing [# (%)]	**--**	--	**24**(26%)	**5**(5%)	**20**(45%)^d,e^
NVP-containing [# (%)]	**--**	--	**15**(16%)	**24**(25%)	**22**(50%)^d,e^
**Body Composition**					
BMI [kg/m2]	**22**(20-26)	**22**(20-26)	**21**(19-24)	**23**(20-25)	**22**(18-25)
WHR [ ]	**0.82**(0.77-0.86)	**0.84**(0.79-0.89)	**0.86**(0.81-0.91)^A^	**0.86**(0.81-0.92)^A^	**0.88**(0.82-1)^A,b^
Skinfold Thickness					
Tricep [mm]	**12.2**(7.6-15.3)	**10.8**(5.4-14.9)^A^	**6.7**(3.9-9.5)^A,B^	**6.7**(4.2-10.0)^A,b^	**8.0**(5.2-10.6)^A^
Bicep [mm]	**6.1**(3.3-8.8)	**6.2**(3.1-9.2)	**3.1**(2.4-5.8)^A,B^	**3.5**(2.4-6.0)^A,B^	**4.4**(2.7-8.1)
Suprailiac [mm]	**9.3**(6.4-13.7)	**7.9**(5.0-13.1)	**6.2**(4.0-8.9)^A,b^	**7.4**(4.3-10.9)^A^	**7.7**(4.0-12.4)
Sub-scapula [mm]	**20.9**(16.0-27.1)	**19.8**(11.7-28.4)	**15.8**(9.9-20.7)^a,b^	**18.4**(11.2-28.2)^d^	**20.2**(9.8-30.4)^d^
Trunk:arm [ ]	**1.6**(1.4-2.0)	**1.8**(1.4-2.2)	**2.1**(1.4-2.8)^a,b^	**2.2**(1.5-3.3)^a,b^	**2.1**(1.5-2.6)^a,b^
Observed change in fat (% observing decrease in fat / % observing increase in fat					
Face [%]	**0%/0%**	**8%/4%**	**27%^A,b^/5%**	**15%^A^/23%** ^**A,b,d**^	**36%^A,b^/11%**
Arms [%]	**0%/0%**	**4%/4%**	**16%^A^/4%**	**8%/13%** ^**A**^	**23%^A^/7%**
Legs [%]	**0%/0%**	**6%/2%**	**22%^A^/5%**	**17%^A^/20%** ^**A,b**^	**25%/7%**
Abdomen [%]	**0%/0%**	**4%/6%**	**1%/32%** ^**A,B**^	**1%/29%** ^**A,B**^	**2%/41%** ^**A,B**^
Chest [%]	**0%/0%**	**4%/0%**	**2%/11%** ^**a,b**^	**1%/9%** ^**a,b**^	**0%/25%** ^**a,b**^
Buffalo hump [%]	**0%/0%**	**4%/0%**	**1%/7%**	**0%/9%** ^**a,b**^	**2%/14%**
Neck [%]	**0%/0%**	**4%/0%**	**3%/5%**	**0%/8%**	**2%/16%**
Body [%]	**0%/8%**	**20%** ^**a**^ **/14%**	**14%^A^/32%** ^**a**^	**13%^a^/42%** ^**A,B**^	**16%/36%**

Continuous variables are reported as median (interquartile range). BMI = body mass index, and WHR = waist-to-hip ratio, mm = millimeters.

A, a = p<0.005 or p<0.05 versus HIV-negative controls, respectively;

B, b = p<0.005 or p<0.05 versus HAART-naive, respectively;

D, d = p<0.005 or p<0.05 versus EFV, respectively;

E, e = p<0.005 or p<0.05 versus NVP, respectively.

### Body Composition and Anthropometric Measurements

No differences were observed in BMI across groups. WHR was elevated in all HAART-treated groups, compared to HIV-negative subjects and in the LPV/r-treated subjects compared to HAART-naïve subjects. Skin-fold thickness measurements yielded multiple differences between HAART treated versus HIV-negative and HAART-naïve subjects. Trunk/Arm skin-fold thickness ratio was significantly higher in all HAART-treated subjects compared to HIV-negative and HAART-naïve subjects. The self-reported fat redistribution followed these trends, with HAART-treated subjects generally reporting decreased fat in their face, arms, and legs, increased fat in the chest, buffalo hump, and neck and increased overall body fat. A significant fraction of NVP-treated subjects also reported increased fat in their face, arms, and legs. Taken together, WHR, skin-fold thickness measurements, and self-reported observations show that HAART-treated subjects in Addis Ababa, Ethiopia exhibit lipodystrophy.

### Blood Pressure, Pulse Wave Analysis, and Pulse Wave Velocity

**Fig 1:**
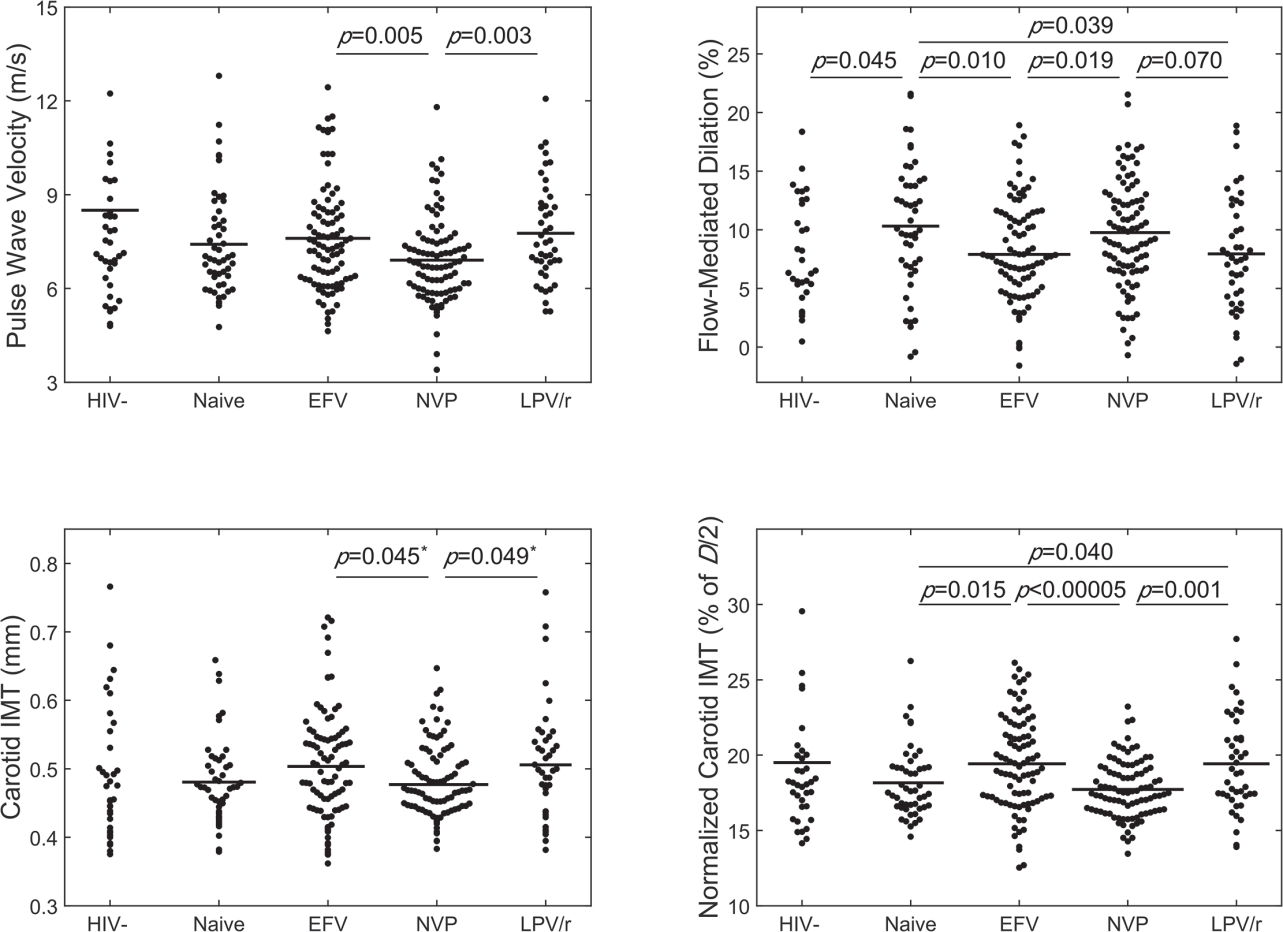
Preclinical markers of atherosclerosis are elevated in EFV-treated and LPV/r-treated subjects compared to HAART-naïve and NVP-treated. Wilcoxon rank sum test *p*-values are shown. *Note: Although the pairwise Wilcoxon rank sum test showed differences in cIMT between EFV- and LPV/r-treated and NVP-treated subject, ANOVA yielded a *p* = 0.12, which is above the defined criteria of *p*<0.05 for statistical significance. When normalized to carotid artery diameter (cIMT-norm = cIMT/(*D*/2), where *D* = carotid diameter), EFV- and LPV/r-treated subjects exhibited significantly increased values compared to NVP-treated and HAART-naïve subject.

The B-SBP was lower in EFV-treated compared to NVP-treated subjects and brachial artery pulse pressure (PP) was lower in EFV-treated compared to NVP- and LPV/r-treated subjects (Table 2). Pulse wave analysis predicted that the aortic PP was higher in the NVP- and LPV/r-treated compared to the HAART-naïve and EFV-treated subjects; aortic PP was higher in HIV-negative compared to HAART-naïve subjects. PWV was significantly lower in NVP-treated compared to EFV-treated and LPV/r-treated subjects, indicating arterial stiffening of the central vasculature (Fig 1).

**Table 2:** Cardiovascular metrics.

End-point	HIV-Negative	HAART Naïve	Efavirenz (EFV)	Nevarapine (NVP)	Lopinavir/r (LPV/r)
	(n=36)	(n=51)	(n=91)	(n=95)	(n=44)
**Cardiovascular Metrics**	** **				
Heart Rate	**66**(64-74)	**72**(65-79)	**70**(65-78)	**71**(65-78)	**69**(63-73)
Blood Pressure					
Brachial SBP [mmHg]	**120**(112-132)	**112**(108-123)	**112**(104-123)^a^	**117**(107-130)^d^	**119**(105-131)
Brachial DBP [mmHg]	**73**(68-81)	**70**(66-79)	**71**(63-78)	**72**(67-82)	**70**(66-79)
Brachial MP [mmHg]	**90**(83-99)	**85**(82-95)	**86**(78-95)	**89**(82-99)	**90**(80-101)
Brachial PP [mmHg]	**46**(40-54)	**43**(37-49)	**42**(38-46)^a^	**45**(39-52)^d^	**45**(38-56)^d^
Pulse Wave Analysis					
Aortic SBP [mmHg]	**110**(101-121)	**103**(98-114)	**103**(96-113)	**109**(98-122)	**112**(97-123)
Aortic DBP [mmHg]	**75**(69-82)	**71**(67-81)	**72**(64-79)	**73**(68-83)	**71**(67-81)
Aortic MP [mmHg]	**90**(83-98)	**85**(82-95)	**86**(78-95)	**89**(82-99)	**90**(80-101)
Aortic PP [mmHg]	**34**(30-42)	**32**(26-38)	**31**(28-36)^a^	**34**(29-42)^b,d^	**34**(31-47)^b,d^
AP [mmHg]	**8**(5-13)	**8**(6-14)	**8**(5-11)	**10**(6-14)	**10**(6-16)
Aix	**27**(15-34)	**25**(20-35)	**26**(20-33)	**27**(20-35)	**28**(21-38)
AIx-75	**26**(12-33)	**25**(19-35)	**26**(17-32)	**27**(19-33)	**28**(19-34)
Pulse Wave Velocity [m/s]	**7.1**(6.3-8.4)	**7.0**(6.3-8.2)	**7.4**(6.3-8.4)	**6.8**(6.0-7.5)^D^	**7.4**(6.8-8.7)^E^
Baseline Diameter [mm]	**3.04**(2.83-3.40)	**2.87**(2.63-3.19)	**2.98**(2.75-3.26)	**3.07**(2.81-3.41)	**2.88**(2.69-2.69)
Dilated Diameter [mm]	**3.29**(3.07-3.56)	**3.22**(2.94-2.94)	**3.22**(2.99-3.51)	**3.33**(3.07-3.76)	**3.20**(2.91-2.91)
FMD [%]	**6.5**(5.0-12.4)	**10.0**(7.0-13.9)^a^	**7.7**(4.8-11.2)^b^	**9.2**(6.6-12.5)^d^	**7.6**(4.5-12.2)b
Common Carotid Artery					
Diameter [mm]	**5.17**(4.94-5.52)	**5.27**(5.02-5.58)	**5.16**(4.92-5.51)	**5.42**(5.08-5.69)^D^	**5.21**(4.90-5.49)^e^
c-IMT [mm]	**0.47**(0.41-0.54)	**0.47**(0.44-0.51)	**0.50**(0.44-0.54)	**0.47**(0.44-0.51)	**0.50**(0.46-0.54)
c-IMT norm [%]	**18.0**(16.4-19.8)	**17.6**(16.6-19.3)	**19.2**(17.2-21.7)^b^	**17.5**(16.3-18.9)^D^	**19.0**(17.4-21.1)^b,E^
Medial stress [kPa]	**55**(49-60)	**55**(49-61)	**50**(45-58)^a,b^	**57**(52-62)^D^	**51**(43-59)^e^
Properties					
Modulus [kPa]	**57**(42-81)	**50**(43-74)	**52**(43-66)	**55**(47-70)	**61**(51-75)
Compliance [MPa-1]	**20**(12-24)	**20**(14-24)	**19**(15-23)	**18**(14-22)	**17**(14-20)
Cyclic strain [ ]	**8.3**(7.4-9.8)	**7.5**(6.7-9.2)	**8.0**(6.8-9.5)	**8.4**(7.0-10.2)	**8.8**(6.9-9.7)
**Blood Sample Analysis**					
Metabolic Parameters					
Glucose [mg/dL]	**93**(85-100)	**88**(83-97)	**94**(88-103)	**89**(83-96)^D^	**89**(83-95)^D^
TC [mg/dL]	**184**(161-230)	**180**(152-206)	**195**(175-229)^b^	**201**(175-239)^B^	**216**(181-240)^b^
TG [mg/dL]	**90**(74-153)	**131**(104-155)	**147**(109-196)^A^	**146**(102-185)^a^	**208**(149-242)^A,B,D,E^
HDL-c [mg/dL]	**53**(46-59)	**43**(39-54)^A^	**46**(40-56)^a^	**49**(41-60)^b^	**45**(38-53)^A,e^
LDL-c [mg/dL]	**106**(91-149)	**108**(91-140)	**123**(101-152)	**126**(95-155)	**129**(108-160)^b^
TC:HDL ratio	**3.5**(3.1-4)	**4.3**(3.7-4.8)^a^	**4.3**(3.6-5.1)A	**4.2**(3.4-5.2)^a^	**4.5**(4.1-5.7)^A,b,e^
Inflammatory Markers					
hs-CRP [ug/mL]	**3.1**(1.5-6.6)	**3.6**(1.8-14.9)	**8.1**(4.2-18.3)^A,b^	**3.9**(2.0-14.8)^d^	**5.4**(2.4-15.9)^a^
s-ICAM [ng/mL]	**521**(454-602)	**711**(574-937)^A^	**651**(509-914)^A^	**657**(526-914)^A^	**651**(504-843)^A^
s-VCAM [ng/mL]	**651**(548-977)	**1114**(768-1952)^A^	**806**(593-1036)^B^	**845**(649-1083)^a,b^	**1011**(606-1409)^a^
Lepitin [ng/mL]	**23**(15-31)	**23**(16-43)	**16**(11-24)^a,B^	**18**(12-27)^b^	**18**(14-31)

Continuous variables are reported as median (interquartile range). bpm = beats per minute, mmHg = millimeters of mergury, SBP = systolic blood pressure, DBP = diastolic blood pressure, MP = mean preasure, PP = pulse pressure, AP = augmentation pressure, AIx = augmentation index, AIx = 75 = augmentation index, normalize to a heart rate of 75 bpm, mm = millimeters, kPa = kiloPascals, TC = total cholesterol, TG = triglycerides, HDL = high density lipoprotein cholesterol, LDL = low density lipoprotein cholesterol, hs-CRP = high sensitivity C-reactive protein, sICAM = soluble intercellular adhesion molecule-1, sVCAM = soluble vascular cell adhesion molecule-1, mg = milligrams, ug = micrograms, ng = nanograms, dL = deciliter, mL = milliliters.

A, a = p<0.005 or p<0.05 versus HIV-negative controls, respectively;

B, b = p<0.005 or p<0.05 versus HAART-naive, respectively;

D, d = p<0.005 or p<0.05 versus EFV, respectively;

E, e = p<0.005 or p<0.05 versus NVP, respectively.

### Flow-mediated dilation (FMD)

The peak FMD was observed at 60 seconds post-occlusion (not shown). FMD at 60 seconds post-occlusion was reduced in EFV-treated and LPV/r-treated compared to HAART-naïve subjects and in EVF-treated compared to NVP-treated subjects.

### Carotid Artery Geometry and Properties

No significant differences in the cIMT were observed across groups. The carotid artery diameter was smaller in NVP- compared to EFV- and LPV/r-treated subjects, both in diastole and systole. When normalized to diameter, however, cIMT^norm^ was significantly increased in EFV- and LPV/r-treated compared to NVP-treated and HAART-naïve subjects. The medial stress was lower in EFV- and LPV/r-treated compared to NVP-treated subjects and lower in EFV-treated compared to HIV-negative and HAART-naïve subjects. No differences were observed in the Peterson modulus, compliance, or cyclic strain over the cardiac cycle across groups.

### Plasma Metabolic Parameters

Fasting glucose was elevated in EFV-treated compared to NVP- and LPV/r-treated subjects. Total cholesterol was elevated in all HAART-treated groups compared to HAART-naïve subjects. Triglycerides were elevated in all HAART-treated groups compared to HIV-negative subjects and in LPV/r-treated subjects compared to all other groups. HDL was lower in HAART-naïve and EFV- and LPV/r-treated compared to HIV-negative subjects and in HAART-naïve and LPV/r-treated compared to NVP-treated subjects. LDL was higher in LPV/r-treated compared to HAART-naïve subjects. Total cholesterol to HDL ratio was elevated in all HAART-treated groups and HAART-naïve compared to HIV-negative subjects and in LPV/r-treated compared to HAART-naïve and NVP-treated subjects.

### Inflammatory Markers and Adipokines

hs-CRP was elevated in EFV-treated compared to HIV-negative, HAART-naïve, and NVP-treated subjects and in LPV/r-treated compared to HIV-negative subjects. sICAM-1 was elevated in all HIV-positive groups, compared to HIV-negative subjects. sVCAM-1 was elevated in HAART-naïve, NVP-treated, and LPV/r-treated compared to HIV-negative subjects and in HAART-naïve subjects compared to EFV- and NVP-treated subjects. Leptin was lower in EFV- and NVP-treated groups compared to HAART-naïve subjects and in EFV-treated compared to HIV-negative subjects.

### Correlation and Regression Analysis

Multivariable regression of data from all HAART treated subjects showed that PWV was associated with age, current EFV and LPV/r used, heart rate, B-SPB, triglycerides, LDL, and hsCRP (Table 3). FMD was associated with age, HIV duration, WHR, and glucose. cIMT was associated with age, current EFV use, skinfold thickness, and blood pressure.

**Table 3:** Correlation analysis and multivariable regression model for PWV, FMD, and cIMT versus key study parameters.

	Pulse Wave Velocity					Flow-mediated Dilation					Carotid Intima-media Thickness				
	Correlation		Multivariable Regression Model			Correlation		Multivariable Regression Model			Correlation		Multivariable Regression Model		
	*ρ*	*P*	*β*	SE(*β*)	*p*	*ρ*	*P*	*β*	SE(*β*)	*p*	*ρ*	*P*	*β*	SE(*β*)	*p*
**Demographics**															
Age	0.531	<0.001	0.0425	0.0151	0.006	-0.163	0.014	-0.00137	0.000564	0.017	0.412	<0.001	0.000240	7.582E-05	0.002
Sex	0.151	0.022	—	—	—	-0.15	0.023	—	—	—	0.164	0.013	—	—	—
**HIV / HAART History**															
CD4 count	-0.154	0.020	—	—	—	0.0429	0.52	—	—	—	0.0314	0.64	—	—	—
log10(VL)	0.066	0.32	—	—	—	-0.011	0.87	—	—	—	0.0531	0.42	—	—	—
Year since HIV+	0.189	0.004	—	—	—	-0.0105	0.87	-0.00357	0.00167	0.035	0.104	0.12	—	—	—
HAART duration	0.228	<0.001	—	—	—	0.0119	0.86	—	—	—	0.140	0.034	—	—	—
Current EFV	0.121	0.067	1.14	0.277	<0.001	-0.0987	0.14	—	—	—	0.0972	0.14	0.0257	0.0122	0.038
Current LPV/r	0.132	0.047	1.05	0.305	<0.001	-0.0471	0.48	—	—	—	0.0744	0.26	—	—	—
**Body Composition**															
BMI	0.107	0.11	—	—	—	0.027	0.68	—	—	—	0.109	0.1	—	—	—
WHR	0.313	<0.001	—	—	—	-0.187	0.005	-0.15	0.0647	0.023	0.171	0.009	—	—	—
Trunk:Arm	0.140	0.034	—	—	—	-0.152	0.022	—	—	—	0.105	0.11	0.00138	0.000517	0.009
**Cardiovascular Metrics**															
Heart Rate	0.204	0.002	0.0278	0.0125	0.028	0.0741	0.26	—	—	—	-0.0711	0.28	—	—	—
B-SBP	0.567	<0.001	0.0332	0.00662	<0.001	-0.147	0.026	—	—	—	0.311	<0.001	6.43E-05	3.14E-05	0.043
AIx75	0.408	<0.001	—	—	—	0.0978	0.14	—	—	—	0.146	0.027	—	—	—
PWV						-0.113	0.088	—	—	—	0.337	<0.001	—	—	—
FMD	-0.113	0.09	—	—	—						-0.111	0.093	—	—	—
cIMT	0.337	<0.001	—	—	—	-0.111	0.093	—	—	—					
**Blood Sample Analysis**															
*Metabolic Parameters*															
Glucose	0.130	0.05	—	—	—	-0.0671	0.31	-0.000615	0.000288	0.035	0.0915	0.17	—	—	—
TG	0.145	0.03	0.0018	0.000888	0.046	-0.084	0.21	—	—	—	0.0274	0.68	—	—	—
HDL-c	0.00364	0.96	—	—	—	0.0049	0.94	—	—	—	0.0954	0.15	—	—	—
LDL-c	0.259	<0.001	0.0063	0.00311	0.046	-0.0212	0.75	—	—	—	0.185	0.005	—	—	—
*Inflammatory Markers*															
VCAM	-0.125	0.06	—	—	—	0.0739	0.27	—	—	—	-0.103	0.12	—	—	—
ICAM	-0.113	0.09	—	—	—	0.134	0.042	—	—	—	-0.118	0.0756	—	—	—
Leptin	-0.0293	0.66	—	—	—	0.127	0.055	—	—	—	-0.0282	0.67	—	—	—
hs-CRP	0.300	<0.001	0.0209	0.00799	0.0106	-0.0531	0.42	—	—	—	0.0820	0.22	—	—	—
				***Adjusted R2 =***	***0*.*604***				***Adjusted R2 =***	***0*.*168***				***Adjusted R2 =***	***0*.*277***

*ρ* = Spearman Rank Correlation Coefficient, *P* = p-value for Spearman correlation, *β* = model parameters, SE(*β*) = standard error of model parameters, *p* = p-value for multivariable regression for that parameter. BMI = body mass index, WHR = waist-to-hip ratio, B-SBP = brachial artery systolic blood pressure, PWV = pulse wave velocity, FMD = flow mediated dilation, cIMT = carotid intima-media thickness, TG = triglycerides, HDL = high density lipoprotein cholesterol, LDL = low density lipoprotein cholesterol, VCAM = soluble vascular cell adhesion molecule, ICAM = souluble intercellular adhesion molecule, hsCRP = C-reactive protein.

## Discussion

### Preclinical Markers of Atherosclerosis

There are numerous studies that associate specific antiretroviral drugs with early onset of atherosclerosis. Protease inhibitors (PI’s) have been the most widely implicated class of antiretroviral drugs associated with atherosclerosis and have been correlated with increased c-IMT [13,14], impaired FMD [15–17], dyslipidemia [10], atherosclerosis [18,19], and myocardial infarction [20], but others have reported contradictory results [21–25]. Non-nucleoside reverse transcriptase inhibitors (NNRTIs) have been associated with elevated cholesterol levels and triglycerides [19], with EFV associated with higher total cholesterol and triglyceride levels than NVP [10]. Less is known about the association between NNRTIs and c-IMT, FMD, and arterial stiffness.

Increased arterial stiffness is a key predictor of future cardiovascular events [26] and is elevated in HIV-positive populations [5,6,27,28]. van Vonderen et al. report decreased distensibility and compliance coefficients in carotid, femoral, and brachial arteries in HIV-positive subjects, with no differences between HAART-naïve and HAART-treated subjects. In a longitudinal study, van Vonderen et al. showed a *decrease* in carotid arterial stiffness, but increase in femoral artery stiffness (i.e., a decrease in distensibility and compliance coefficients) after 24 months of AZT+3TC+LPV/r treatment, with similar trends in NVP+LPV/r treatment [6]. The current report shows that central arterial stiffness measured via PWV was higher in EFV- and LPV/r-treated subjects, compared to NVP-treated subjects; however, no differences were observed in the carotid artery elastic modulus, compliance, or the distensibility or compliance coefficients (not shown) across groups. Note that arterial stiffening in aorta, but not CCA’s, is consistent with recent findings in a mouse model of AZT treatment [29].

Arterial stiffness appears to increase with HAART [5,6,28] and is associated with immune cell activation and senescence [27] and may be independent of lipodystrophy [30]. In the current study, PWV was associated with EFV and LPV/r use, traditional cardiovascular risk factors (age, heart rate, B-SBP), dyslipidemia (triglycerides and LDL), and systemic inflammation (hsCRP). Taken together, the current study and previous studies from the literature suggest that both HIV infection and HAART play a role in arterial stiffening, with differing effects at different locations in the vasculature.

Impaired brachial artery FMD is a non-invasive indicator of endothelial dysfunction and is associated with elevated cardiovascular risk in the general population [31]. Impaired FMD is also widely reported in HIV-infected populations and appears to be associated with both viral RNA levels [32,33] and HAART [8,9,11,34]. Although not significantly different than pre-HAART values, Gupta *et al*. reports a significant decrease in FMD in EFV-treated compared to PI-treated subjects after 12-months of treatment [34]. Impaired FMD has been associated with lipodystrophy [35]; the association between FMD and dyslipidemia with HIV, however, is debatable [8]. Our data show that EFV- and LPV/r-treated subject exhibit lower FMD, compared to NVP-treated and HAART-naïve subjects. Multivariant regression shows that FMD was associated with age, duration of HIV, WHR, and fasting blood glucose levels.

Increased cIMT has been widely reported in HIV-infected populations [6,7,32,36–39], is strongly associated with all-cause death in HIV patients [40], and appears to be associated with both HIV infection and HAART regimen [36,38]. PI’s have been implicated [4] as a mediator of increased cIMT and cIMT has been associated with immune cell activation [41,42], systemic inflammation [40,42,43], and Framingham risk [44]. Our data show nominally higher cIMT in EFV- and LPV/r-treated subject; however, increased cIMT only becomes significant when normalized to *D*/2. Multivariant regression showed that cIMT was associated with current EFV use, traditional cardiovascular risk factors (age and B-SBP), and lipodystrophy (Trunk:Arm).

Our results show elevated markers of atherosclerosis in EFV- and LPV/r-treated subjects, compared to HAART-naïve and NVP-treated groups. However, there are several compounding factors at play that contribute to this observation. First, the years since HIV diagnosis in the HAART-treated group is nearly double that of the HIV-naïve group. however, the NVP group had a longer duration of HIV infection, compared to the EFV group. Second, more subjects in the EFV- and LPV/r-treated groups were taking an NRTI-backbone of TDF+3TC, compared to the NVP-treated group, which had more subjects taking AZT+3TC. The role of AZT in arterial stiffening, endothelial dysfunction, and cIMT is well documented [45–47]. We included TDF and AZT in early iterations of our multivariable regression model; due to their low association with arterial stiffness, cIMT, and FMD, TDF and AZT were considered key predictors of these markers and were subsequently eliminated from the final model. Clinical evidence for atherosclerosis associated with NRTIs is often indirect and difficult to define because NRTIs are typically not prescribed as monotherapy and cardiovascular effects are often attributed to other components of HAART (namely PIs). Nevertheless, recent exposure to abacavir, didanosine, and tenofovir in adult populations was associated with increased risk of heart failure [48–51]; however, these findings are controversial [52]. Abacavir has been associated with impaired FMD [53]. Experimental models, including those from our group, clearly show that AZT, induces endothelial dysfunction [45,54] and increased c-IMT and arterial stiffness [55].

Data from the literature suggest that HIV, independent of HAART, can induce cardiovascular disease [56–58]. Post-mortem analyses reveal coronary arteriopathy [59,60], pulmonary arteriopathy [60], major atherosclerotic lesions [60,61] and cerebral aneurysms [62] in HIV-positive patients not treated with HAART. A long-term multi-institution analysis concluded that cessation of HAART in HIV-positive patients increased their short-term risk of developing cardiovascular disease [56]. Viral load correlated inversely with endothelium-dependent FMD without any relation to HAART regimens [63]; thus, viral load is a significant predictor of impaired FMD [25]. HIV infected, HAART-naïve patients exhibited elevated c-IMT and impaired FMD [57] and HIV-infected children had significantly reduced FMD and increased arterial stiffness of the carotid artery compared to non-infected children, with no significant differences between HAART treated and HAART naïve subjects [64]. Lorenz et al. concluded that both HAART and HIV-infection are independent risk factors for the development of atherosclerosis in adults [65]. They show that c-IMT of the carotid bifurcation was 25% higher in HIV positive / HAART-naïve patients compared to uninfected controls, but observed significantly greater c-IMT of the carotid bifurcation and the common carotid artery due to HAART treatment in HIV positive subjects. Although c-IMT, FMD, and arterial stiffening are affected by HIV infection, it appears that HIV infection only introduces mild dyslipidemia [57,66,67].

### Metabolic Disorders

Alterations in plasma lipid profiles have been reported in both HAART-naïve and HAART-treated subjects that are characterized by increased TG’s and decreased HDL; increased total cholesterol and LDL are also reported and PI-containing regimens are generally associated with a less favorable lipid profile compared to PI-sparing regimens [10,68]. Our results are consistent with these observations, with both HAART-naïve and HAART-treated subjects showing less favorable lipid profiles than HIV-negative subjects, and PI-treated subjects exhibiting worse lipid profiles than other HIV-positive groups.

HIV-associated lipodystrophy is characterized by peripheral lipoatrophy and central lipohypertrophy, and are often associated with disorders in glucose metabolism, insulin resistance, dyslipidemia, altered cytokine and adipokine production, and markers of cardiovascular disease [1,69]. Self-reported observations of fat redistribution in the present study suggest similar trends, with a significant fraction of HAART subjects reporting observed decreases in peripheral fat and an increase in central fat. In addition, NVP-treated subjects reported increase in fat in their face, arms, and legs. Skin-fold thickness measurements support these self-reports, with EFV-, NVP-, and LPV/r-treated subjects having higher Trunk/arm ratios compared to HIV-negative and HAART-naïve subjects. Note that the trunk skin-fold thickness measurement did not suggest central lipohypertrophy, as superiliac and sub-scapula values were generally lower or not significantly different in HAART-treated compared to non-HAART-treated subjects.

### Inflammatory Markers & Adipokines

hsCRP is a key predictor of future cardiovascular events in the general population [70–72], is widely reported to be elevated in HIV populations [7,39,43,73–79] (noting contradictory reports [27,32,80]), and is associated with all-cause death in HIV patients [40]. Most studies show little or no HAART dependency [6,33,34,76,81] and hsCRP appears to be independent of lipodystrophy [35]. Our results show that hsCRP is elevated in EFV-treated subjects compared to HIV-negative, HAART-naïve, and NVP-treated subjects and elevated in LPV/r-treated subjects compared to HIV-negative subjects and that hsCRP is a key predictor of PWV.

sVCAM-1 and sICAM-1 are plasma biomarkers that have been associated with the initiation and progression of atherosclerosis [82] and are commonly associated with HIV infection [76,79,80,83]. Following initiation of HAART, values decrease from pre-HAART levels, but remain higher than HIV-negative controls [6,34,76,84]. Our results are consistent with these findings, with sVCAM-1 and sICAM-1 levels elevated in HAART-naïve and, to a lesser degree, in HAART-treated subjects compared to HIV-negative subjects, but no correlation between sVCAM-1 or sICAM-1 levels and PWV, FMD, or cIMT were found.

Adipokines such as adiponectin and leptin have been studied intensively for their relation to obesity, insulin resistance, and hyperglycemia and as potential proatherogenic mediators [85,86]. Low leptin and adiponectin levels are more common in persons with HIV and correlate with lipodystrophy [35,73,79,87]. Our results show reduced leptin levels with HAART treatment, but no correlation between leptin levels and PWV, FMD, or cIMT were found.

### Study Limitations

This study has several limitations. The cross-sectional design of this study does not allow for a causal relationship between specific antiretroviral drugs and the markers studied. As is often the case, there were challenges in recruiting a representative HIV-negative control group. Controls were recruited by word of mouth at Black Lion hospital, and some recruits may have been referred from the Cardiac clinic. This may explain, in part, why the preclinical markers of atherosclerosis were nominally, albeit not significantly higher (except for FMD), in the controls versus NVP-treated and HAART-naïve groups. Although the normalization of the cIMT was motivated to normalize values across a broad range of patients and showed significant differences across groups, although the more traditional ‘raw’ cIMT data followed similar trends, the raw data did not show significant differences. Also, the cIMT images were taken from a single profile view, rather than the average of several orthogonal views and the FMD protocol did not include nitroglycerine to quantify the endothelial independent vasodilation. Taken together, we believe these limitations do not significantly bias the conclusions of this study.

## Conclusion

This report suggests that current EFV and LPV/r may play a role in the development of HIV-associated preclinical markers of atherosclerosis. The data presented herein are consistent with previous results, in that HAART-treated subjects exhibit elevated cholesterol, TC:HDL ratio, and triglycerides and elevated markers of inflammation. What is surprising, however, is that although NVP-treated subjects have similar traditional risk factors (e.g., dyslipidemia, lipodystrophy) compared to EFV-treated subjects, NVP-treated subjects appear to be protected from elevations in preclinical markers of atherosclerosis. These data suggest that the early on-set atherosclerosis may arise from inflammatory mechanisms or mechanisms that are distinct from traditional risk factors and delineate the need for additional longitudinal studies to establish a causal relationship between EFV- and LPV/r-treatment and atherosclerosis in this population.

## Supporting Information

S1 File Includes results on smoking and alcohol use, personal and familial history of heart attack, angina, or stroke, kidney disease, diabetes, or lipid disorders, and complete blood count results. (DOCX)
