# Supplementary material for: Current Efavirenz (EFV) or Ritonavir-Boosted Lopinavir (LPV/r) Use Correlates with Elevate Markers of Atherosclerosis in HIV-Infected Subjects in Addis Ababa, Ethiopia
Source: PLoS One. 2015 Apr 27;10(4):e0117125. doi: 10.1371/journal.pone.0117125 (PMC4411122; doi:10.1371/journal.pone.0117125)
Supplement: S1 File — (DOCX) [file pone.0117125.s001.docx]

**Supplemental Materials**

**Additional Participant Characteristics**

Less than 3% of the participants in any group reported being current cigarette smokers and less than 6% of the participants in any group report drinking more than 4 drinks per week (Table A1 in Supplemental Material). Less than 10% and 15% of the subjects from any group reported a family history and personal history, respectively, of heart attack, angina, or stroke, kidney disease, diabetes, or lipid disorders.

**Complete Blood Count**

No differences were observed in the number of percentages of white blood cells (WBC) or distribution of WBC classes across groups. Red blood cell markers suggest a shift towards a more anemic state in HAART-treated and, to a lesser degree, HAART-naïve subjects. Namely, red blood cell count was lower and mean corpuscular volume, mean corpuscular hemoglobin, and red blood cell distribution width-standard deviation were higher in all HAART-treated groups compared to HIV-negative and HAART-naïve subjects and hemoglobin and hematocrit were lower in all HAART-treated groups and HAART-naïve subjects compared to HIV-negative subjects. (Note: *p* = 0.06 comparing hemoglobin between EFV-treated and HIV-negative subjects.) Platelet count and plateletcrit were elevated in EFV-treated subjects compared to all other HIV-positive groups; platelet count was also higher in EFV-treated and plateletcrit lower in NVP-treated subjects compared to HIV-negative subjects.

**TABLE CAPTION**

**TABLE S1: Lifestyle, personal and familial history of CVD, and complete blood count (CBC) analysis**. Continuous variables are reported as median (interquartile range). HA = heart attack, WBC = white blood cells, Neu = neutrophil, Lym = lymphocytes, Mon = monocytes, Eos = eosinophil granulocytes, Bas = basophil granulocytes, RBC = red blood cells, HGB = hemoglobin, HCT = hematocrit, MCV = mean corpuscular volume, MCH = mean corpuscular hemoglobin, MCHC = mean corpuscular hemoglobin concentration, RDW-CV = red blood cell distribution width-coefficient variation, RDW-SD = red blood cell distribution width-standard deviation, PLT = platelets, MPV = mean platelet volume, PDW = platelet distribution width, and PCT = plateletcrit, g = grams, mg = milligrams, ug = micrograms, ng = nanograms, pg = pictograms, dL = deciliter, mL = milliliters, uL = microliters, fL = femtoliters. A, a = p<0.005 or p<0.05 versus HIV-negative controls, respectively; B, b = p<0.005 or p<0.05 versus HAART-naive, respectively; D, d = p<0.005 or p<0.05 versus EFV, respectively; E, e = p<0.005 or p<0.05 versus NVP, respectively.

**TABLE S1:**

|  |  |  |  |  |  |  |  |  |  |  |  |  |  |  |  |  |  |  |  |  |  |  |
| --- | --- | --- | --- | --- | --- | --- | --- | --- | --- | --- | --- | --- | --- | --- | --- | --- | --- | --- | --- | --- | --- | --- |
|  | **End-point** | | | **HIV-Negative** | | | **HAART Naïve** | | | | **Efavirenz (EFV)** | | | | **Nevarapine (NVP)** | | | | **Lopinavir/r (LPV/r)** | | | |
|  |  |  |  |  | **(n=** | **36)** |  | **(n=** | **51)** |  |  | **(n=** | **91)** |  |  | **(n=** | **95)** |  |  | **(n=** | **44)** |  |
|  |  |  |  |  |  |  |  |  |  |  |  |  |  |  |  |  |  |  |  |  |  |  |
|  |  |  |  |  |  |  |  |  |  |  |  |  |  |  |  |  |  |  |  |  |  |  |
|  | **Lifestyle** | | |  |  |  |  |  |  |  |  |  |  |  |  |  |  |  |  |  |  |  |
|  |  | Lifetime >100 Cigarettes [%] | | **6%** | | | **4%** | | |  | **8%** | | |  | **6%** | | |  | **11%** | | |  |
|  |  | Current smoker [%] | | **3%** | | | **2%** | | |  | **0%** | | |  | **2%** | | |  | **0%** | | |  |
|  |  | Consume ≥1 drinks/month [%] | | **75%** | | | **63%** | | |  | **46%** | | | **^a^** | **44%** | | | **^a^** | **48%** | | |  |
|  |  | Consume >4 drink/week [%] | | **3%** | | | **6%** | | |  | **3%** | | |  | **2%** | | |  | **2%** | | |  |
|  | **Personal History of CVD** | | |  |  |  |  |  |  |  |  |  |  |  |  |  |  |  |  |  |  |  |
|  |  |  | HA, Angina, or Stroke [%] | **3%** | | | **4%** | | |  | **4%** | | |  | **2%** | | |  | **0%** | | |  |
|  |  |  | Kidney Disease [%] | **3%** | | | **8%** | | |  | **7%** | | |  | **5%** | | |  | **2%** | | |  |
|  |  |  | Lipid Disorders [%] | **0%** | | | **4%** | | |  | **5%** | | |  | **4%** | | |  | **9%** | | |  |
|  | **Family History of CVD** | | |  |  |  |  |  |  |  |  |  |  |  |  |  |  |  |  |  |  |  |
|  |  |  | HA, Angina, or Stroke [%] | **0%** | | | **6%** | | |  | **4%** | | |  | **9%** | | |  | **14%** | | |  |
|  |  |  | Kidney Disease [%] | **0%** | | | **2%** | | |  | **0%** | | |  | **3%** | | |  | **5%** | | | **^d^** |
|  |  |  | Diabetes [%] | **6%** | | | **4%** | | |  | **12%** | | |  | **16%** | | |  | **11%** | | |  |
|  |  |  | Lipid Disorders [%] | **0%** | | | **0%** | | |  | **0%** | | |  | **0%** | | |  | **2%** | | |  |
|  | **Blood Sample Analysis** | | |  |  |  |  |  |  |  |  |  |  |  |  |  |  |  |  |  |  |  |
|  |  | Complete Blood Count | |  |  |  |  |  |  |  |  |  |  |  |  |  |  |  |  |  |  |  |
|  |  |  | WBC [10^3^/uL] | **6.01** | (4.55 - | 6.71) | **5.40** | (4.29 - | 6.49) |  | **5.69** | (4.12 - | 6.96) |  | **4.86** | (3.83 - | 6.48) |  | **4.95** | (4.06 - | 5.89) |  |
|  |  |  | Neu [%] | **56.6** | (46.6 - | 63.3) | **51.3** | (42.9 - | 60.5) |  | **54.7** | (41.6 - | 63.9) |  | **50.8** | (42.3 - | 59.6) |  | **52.8** | (43.3 - | 62.7) |  |
|  |  |  | Lym [%] | **34.4** | (26.3 - | 45.1) | **34.9** | (28.4 - | 45.8) |  | **33.9** | (26.9 - | 44.6) |  | **39.2** | (30.1 - | 47.7) |  | **37.2** | (29.9 - | 41.3) |  |
|  |  |  | Mon [%] | **3.4** | (2.6 - | 5.6) | **4.3** | (2.8 - | 6.0) |  | **4.2** | (2.9 - | 6.6) |  | **4.9** | (2.8 - | 6.7) |  | **5.5** | (3.6 - | 7.4) |  |
|  |  |  | Eos [%] | **3.5** | (2.1 - | 5.3) | **3.9** | (2.0 - | 6.9) |  | **3.5** | (1.6 - | 7.0) |  | **3.7** | (2.0 - | 6.1) |  | **4.1** | (2.4 - | 6.1) |  |
|  |  |  | Bas [%] | **0.5** | (0.4 - | 0.8) | **0.4** | (0.3 - | 0.6) |  | **0.5** | (0.3 - | 0.6) |  | **0.5** | (0.3 - | 0.7) |  | **0.6** | (0.4 - | 0.8) |  |
|  |  |  | MID [%] | **8.0** | (6.2 - | 11.1) | **9.7** | (7.0 - | 13.8) |  | **9.1** | (7.3 - | 13.4) |  | **8.9** | (6.8 - | 13.3) |  | **10.5** | (8.5 - | 13.4) |  |
|  |  |  | Neu # [10^3^/uL] | **3.32** | (2.01 - | 4.48) | **2.76** | (1.80 - | 3.86) |  | **2.90** | (1.83 - | 4.28) |  | **2.29** | (1.71 - | 3.17) |  | **2.36** | (1.70 - | 3.18) |  |
|  |  |  | Lym # [10^3^/uL] | **2.01** | (1.59 - | 2.31) | **1.94** | (1.39 - | 2.42) |  | **1.85** | (1.50 - | 2.20) |  | **1.83** | (1.37 - | 2.31) |  | **1.70** | (1.37 - | 2.32) |  |
|  |  |  | Mon # [10^3^/uL] | **0.19** | (0.15 - | 0.31) | **0.22** | (0.17 - | 0.31) |  | **0.22** | (0.14 - | 0.36) |  | **0.21** | (0.14 - | 0.35) |  | **0.28** | (0.15 - | 0.35) |  |
|  |  |  | Eos # [10^3^/uL] | **0.21** | (0.10 - | 0.35) | **0.22** | (0.09 - | 0.37) |  | **0.17** | (0.09 - | 0.37) |  | **0.17** | (0.07 - | 0.29) |  | **0.18** | (0.11 - | 0.33) |  |
|  |  |  | Bas # [10^3^/uL] | **0.03** | (0.02 - | 0.04) | **0.02** | (0.0 - | 0.0) |  | **0.02** | (0.01 - | 0.03) |  | **0.02** | (0.01 - | 0.04) |  | **0.03** | (0.02 - | 0.03) |  |
|  |  |  | MID [#] | **0.46** | (0.37 - | 0.64) | **0.49** | (0.35 - | 0.75) |  | **0.50** | (0.34 - | 0.71) |  | **0.41** | (0.30 - | 0.67) |  | **0.55** | (0.37 - | 0.69) |  |
|  |  |  | RBC [10^6^/uL] | **5.14** | (4.76 - | 5.31) | **4.84** | (4.43 - | 5.29) |  | **4.40** | (4.05 - | 4.67) | **^A,B^** | **4.07** | (3.68 - | 4.57) | **^A,B,D^** | **4.33** | (3.98 - | 4.53) | **^A,B^** |
|  |  |  | HGB [g/dL] | **15.3** | (14.6 - | 16.3) | **14.9** | (13.8 - | 15.7) | **^a^** | **15.0** | (14.1 - | 15.8) |  | **15.0** | (14.1 - | 15.7) | **^a^** | **14.4** | (13.6 - | 15.2) | **^A,d,e^** |
|  |  |  | HCT [%] | **44.5** | (43.2 - | 47.9) | **43.5** | (40.2 - | 46.1) | **^a^** | **43.1** | (41.2 - | 45.0) | **^A^** | **42.6** | (40.5 - | 45.6) | **^A^** | **42.5** | (40.0 - | 44.4) | **^A^** |
|  |  |  | MCV [fL] | **89.3** | (86.6 - | 92.4) | **88.7** | (85.8 - | 92.5) |  | **97.8** | (91.6 - | 105.2) | **^A,B^** | **104.6** | (97.5 - | 113.8) | **^A,B,D^** | **98.4** | (95.1 - | 103.6) | **^A,B,E^** |
|  |  |  | MCH [pg] | **30.5** | (29.7 - | 31.6) | **30.2** | (29.1 - | 31.6) |  | **34.0** | (32.0 - | 37.1) | **^A,B^** | **36.5** | (33.3 - | 40.2) | **^A,B,D^** | **33.2** | (32.2 - | 36.6) | **^A,B,E^** |
|  |  |  | MCHC [g/dL] | **34.4** | (33.9 - | 34.6) | **34.1** | (33.3 - | 34.4) |  | **34.5** | (33.6 - | 35.3) | **^b^** | **34.7** | (33.8 - | 35.4) | **^a,B^** | **34.2** | (33.3 - | 34.8) | **^d,E^** |
|  |  |  | RDW-CV [%] | **12.2** | (12.0 - | 12.5) | **12.6** | (12.2 - | 13.2) | **^a^** | **12.9** | (12.2 - | 13.9) | **^A^** | **12.1** | (11.6 - | 12.8) | **^B,D^** | **12.6** | (12.0 - | 13.4) | **^e^** |
|  |  |  | RDW-SD [fL] | **45.0** | (43.4 - | 46.6) | **46.1** | (44.6 - | 47.9) |  | **50.0** | (47.6 - | 52.7) | **^A,B^** | **51.2** | (47.5 - | 55.0) | **^A,B^** | **49.6** | (47.4 - | 54.5) | **^A,B^** |
|  |  |  | PLT [103/uL] | **254** | (213 - | 282) | **247** | (206 - | 275) |  | **273** | (233 - | 331) | **^a,B^** | **243** | (197 - | 279) | **^D^** | **246** | (210 - | 281) | **^D^** |
|  |  |  | MPV [fL] | **9.5** | (9.0 - | 10.2) | **9.1** | (8.7 - | 9.9) |  | **8.9** | (8.0 - | 9.6) | **^A^** | **8.8** | (8.3 - | 9.7) | **^a^** | **9.1** | (8.7 - | 10.1) |  |
|  |  |  | PDW | **16.0** | (15.7 - | 16.2) | **15.9** | (15.8 - | 16.1) |  | **15.8** | (15.1 - | 16.1) |  | **15.8** | (12.2 - | 16.2) |  | **15.9** | (15.6 - | 16.2) |  |
|  |  |  | PCT [%] | **0.238** | (0.206- | 0.272) | **0.220** | (0.194- | 0.257) |  | **0.250** | (0.210- | 0.287) | **^B^** | **0.209** | (0.181- | 0.245) | **^a,D^** | **0.218** | (0.197- | 0.249) | **^D^** |
|  |  |  |  |  |  |  |  |  |  |  |  |  |  |  |  |  |  |  |  |  |  |  |
